# Localizing the lipid products of PI3Kγ in neutrophils

**DOI:** 10.1016/j.jbior.2015.10.005

**Published:** 2016-01

**Authors:** Laura Norton, Yvonne Lindsay, Arnaud Deladeriere, Tamara Chessa, Hervé Guillou, Sabine Suire, John Lucocq, Simon Walker, Simon Andrews, Anne Segonds-Pichon, Oliver Rausch, Peter Finan, Takehiko Sasaki, Cheng-Jin Du, Till Bretschneider, G. John Ferguson, Phillip T. Hawkins, Len Stephens

**Affiliations:** a The Signalling Department, The Babraham Institute, The Babraham Research Campus, Cambridge, CB22 3AT, United Kingdom; b Division of Molecular Physiology, College of Life Sciences, University of Dundee, Dundee, DD1 5EH, UK; c Division of Cell Biology and Immunology, College of Life Sciences, University of Dundee, Dundee, DD1 5EH, UK; d Laboratoire de Pharmacologie et Toxicologie, INRA UR66, Toulouse, France; e The Imaging Facility, The Babraham Institute, The Babraham Research Campus, Cambridge, CB22 3AT, UK; f The Bioinformatics Group, The Babraham Institute, The Babraham Research Campus, Cambridge, CB22 3AT, UK; g UCB, Allée de la Recherche, 60 1070 Brussels, Belgium; h Developmental and Molecular Pathways, Novartis Institutes for BioMedical Research, 250 Massachusetts Avenue, Cambridge, MA 02139, USA; i Department of Pathology and Immunology, Akita University School of Medicine, 1-1-1 Hondo, Akita 010-8543, Japan; j Warwick Systems Biology Centre, University of Warwick, UK

**Keywords:** PI3K, Neutrophil, Polarization, PI3K, Phosphoinositide 3-kinase, eGFP, enhanced Green Fluorescent Protein, PH, Pleckstrin homology, EM, Electron Microscopy, fMLP, formylated-Met-Leu-Phe, GPCR, G-Protein Coupled Receptor, PKB, Protein Kinase B (also called Akt), TAPP-1, TAndem PH domain containing Protein-1

## Abstract

Class I phosphoinositide 3-kinases (PI3Ks) are important regulators of neutrophil migration in response to a range of chemoattractants. Their primary lipid products PtdIns(3,4,5)P_3_ and PtdIns(3,4)P_2_ preferentially accumulate near to the leading edge of migrating cells and are thought to act as an important cue organizing molecular and morphological polarization. We have investigated the distribution and accumulation of these lipids independently in mouse neutrophils using eGFP-PH reportersand electron microscopy (EM).

We found that authentic mouse neutrophils rapidly polarized their Class I PI3K signalling, as read-out by eGFP-PH reporters, both at the up-gradient leading edge in response to local stimulation with fMLP as well as spontaneously and randomly in response to uniform stimulation. EM studies revealed these events occurred at the plasma membrane, were dominated by accumulation of PtdIns(3,4,5)P_3_, but not PtdIns(3,4)P_2_, and were dependent on PI3Kγ and its upstream activation by both Ras and Gβγs.

## 1 Introduction

Neutrophils are key players in the innate anti-bacterial and fungal defence mechanisms of mammals. They are capable of exiting the circulation in the proximity of local infections or tissue damage and chemotaxing towards the epicentre of the inflammatory response. These processes ready the neutrophils to phagocytose and destroy pathogens or dead cells and hence contribute to the resolution of the problem. Conversely, a number of inflammatory diseases are, in part, driven by excessive, inappropriate neutrophil infiltration (Cowburn et al., 2008, Lee et al., 2003 {Cowburn et al., 2008 #776).

Many further cell types can chemotax and the process is of very wide biological importance. Neutrophil chemotaxis towards inflammatory ligands for GPCRs, such as fMLP, can be studied in vitro and is amongst the most efficient in terms of cell speed and directionality and as a result has been subject to intense investigation aimed at defining the underlying molecular mechanisms and principles. This work has identified a large number of intracellular signals and/or proteins upon which neutrophil chemotaxis can depend. Amongst these phosphoinositide 3-kinases (PI3Ks) (Hirsch et al., 2000, Li et al., 2000, Sasaki et al., 2000) and Rac-family GTPases (Roberts et al., 1999, Servant et al., 2000, Weiner et al., 2002) have been implicated repeatedly but other key regulators include Ca^2+^ (Evans and Falke, 2007) and p38 MAPKs (Heit et al., 2002). Although PtdIns5P has been implicated in cell migration (Viaud et al., 2014 #3612) there is no evidence establishing that it has a role in neutrophil chemokinesis (Bulley et al., 2015).

Many reports have contributed to a picture of chemotaxis in which Class I PI3K signalling takes a central role (Meili et al., 1999, Parent et al., 1998, Servant et al., 2000). Class I PI3Ks, activated in response to fMLP and drive accumulation of PtdIns(3,4,5)P_3_ and PtdIns(3,4)P_2_ at the cell surface (Stephens et al., 1991, Traynor-Kaplan et al., 1988). This has fundamental event has been described using a variety of approaches to measure phosphoinositides, including relatively recently evolved mass-spec-based methods that can resolve different molecular species of PtdIns(3,4,5)P_3_ (Kielkowska et al., 2014). The increase in PtdIns(3,4,5)P_3_ and PtdIns(3,4)P_2_ in the peripheral membrane drives translocation and activation of PI3K effectors including PKB (AKT) and important modulators of the cytoskeleton, such as ARAP3 and DOCK2 (Krugmann et al., 2002, Kunisaki et al., 2006). Crucially, this process can polarise spontaneously, in the presence of uniform fMLP, to generate a leading-edge enriched in PtdIns(3,4)P_2_/(3,4,5)P_3_ (Servant et al., 2000) and a range of other signalling molecules (some targeted by PI3K activity directly) and of characteristic, dynamic morphology. This polarization is important for the neutrophils to move efficiently and hence for chemotaxis, and it is substantially, but not entirely, dependent on Class I PI3K signalling (Ferguson et al., 2007, Niggli and Keller, 1997, Nishio et al., 2007). Remarkably, by-passing receptor activation by direct, uniform shuttling of heterologous PtdIns(3,4,5)P_3_ (Derman et al., 1997, Niggli, 2000, Weiner et al., 2002) or homogenous “chemical” activation of a Class I PI3K construct in neutrophil-like cells (Inoue and Meyer, 2008) leads to spontaneous polarization of both their morphology and endogenous PtdIns(3,4)P_2_/(3,4,5)P_3_, suggesting that PI3K signalling is a core player in an auto-catalytic symmetry-breaking circuit. It has been argued this is a keystone, ubiquitous principle in Eukaryotic chemotaxis. Several issues remain unclear. Firstly, what happens to PtdIns(3,4)P_2_ dynamics? Does PtdIns(3,4)P_2_ simply “follow” PtdIns(3,4,5)P_3_ signals in living neutrophils or does it show evidence of independent regulation and hence function. Secondly, what is the precise cellular location of the pools of PtdIns(3,4,5)P_3_ and/or PtdIns(3,4)P_2_? Are these lipids in the plasma membrane or peripheral endo-membranes? Given the morphological characteristics of neutrophils, with very thin layers of cytoplasm unevenly spread around large lobed nuclei, this has remained unclear.

In this work we have addressed the identity and EM-level localization of the lipid products of PI3K activity accumulated in migrating mouse neutrophils.

## 2 Methods and materials

### 2.1 Materials

All materials used were of the lowest endotoxin level available and were purchased from Sigma-Aldrich unless stated otherwise. PI3K inhibitors have been previously described (Condliffe et al., 2005) (Condliffe et al., 2005).

### 2.2 Mouse strains

The eGFP-PH-PKB mouse strain has been previously described (Nishio et al., 2007) and was bred against p110γ^−/−^ (Hirsch et al., 2000); p101^−/−^ (Suire et al., 2006) and p110γ^DASAA/DASAA^ (Suire et al., 2006) mice. Animals were housed in high health status isolators.

### 2.3 Mouse neutrophil isolation

Was essentially as described (Ferguson et al., 2007). Neutrophil purity was assessed by cytospin.

## 3 Chemotaxis assays

### 3.1 Micropipette chemotaxis assay

Were conducted as described (Ferguson et al., 2007), micropipettes contained 10 μM fMLP. Wortmannin (250 nM) was applied for 10 min while neutrophils settled on the coverslip, prior to stimulation.

### 3.2 Bath application fMLP- chemokinesis assay

Neutrophils (1.5 ml, 1.25 × 10^5^/ml) were settled onto a glass coverslip (22 mm, thickness no 1.5, VWR) held in an imaging ring at 37 °C for 5 min fMLP or peroxy-vanadate (final concentrations 1 μM or 12 mM) were added directly onto cells.

### 3.3 Assessing eGFP-PH-PKB reporter distribution

Polar plots were derived from movies of eGFP-PH-PKB neutrophil chemotaxis (Anagraph, Simon Andrews). QuimP software ((Dormann et al., 2002), Garching Innovation) was used to determine the ratio of membrane intensity to mean cytoplasmic intensity in every frame of the movie at 100 sites around the periphery of the neutrophil. The ratios from individual cells were translated onto a common pseudo-coloured scale where red indicates a high ratio and hence reporter concentration. Each individual cells intensity measurements for one frame were mapped onto a circle and sequential frames were then combined concentrically, with the first frame at the centre. Plots were aligned so that the fMLP containing micropipette (originally 20 μm from the cell) was positioned vertically below.

Production of Retrovirus, harvesting of foetal liver Cells, transduction of foetal liver cells and the injection of foetal liver cells into recipient mice.

#### 3.3.1 Harvesting foetal liver cells

Following timed matings pregnant mice were sacrificed at day 14.5 and foetal liver cells were harvested in Stempro-34 serum-free medium (Invitrogen) (supplemented with provided StemPro-nutrient supplement and 1% penicillin/streptomycin/amphotericin B). Cells were collected, resuspended at 1.7 × 10^7^/ml in FBS +10% DMSO and slowly frozen to −80 °C.

#### 3.3.2 Transfection of Plat-E cells to produce retrovirus

Plat-E cells were cultured in DMEM high glucose with l-glutamine and pyruvate (Gibco) (supplemented with 10% FBS and 1% penicillin/streptomycin) at 37 °C with 5% CO_2_. Plat-E cells were transfected using Lipofectamine 2000 (Invitrogen, as per manufacturer's instructions). Briefly, retroviral transfer DNA (16.8 μg) and Lipofectamine 2000 (50.4 μl) were incubated in 4.2 ml of Optimem-I with Glutamax (Invitrogen) for 30 min at RT. After incubation Plat-E cells were resuspended at 1.19 × 10^6^ in 14 ml of DMEM high glucose with l-glutamine and pyruvate (supplemented with 10% FBS) and mixed with the Lipofectamine 2000/DNA mix. Cells were plated into a 14 cm tissue culture dish and incubated at 37 °C with 5% CO_2_. After 24 h media was replaced with 9 ml of complete Stempro-34 media and incubated for a further 24 h. Viral supernatant was harvested, centrifuged (3000× *g*, 5 min) and 0.45 μm filtered (Millipore). Supernatant was then slowly frozen on dry ice before being stored at −80 °C.

#### 3.3.3 Reconstitution using foetal liver

Foetal liver cells were rapidly thawed and resuspended at 5 × 10^5^/ml in complete Stempro-34 serum-free medium (Invitrogen) containing a cocktail of cytokines (mSCF final concentration 100 ng/ml; TPO 100 ng/ml; hIL-6 1 ng/ml; mIL3 6 μg/ml; and Flt3L 20 ng/ml) (Peprotech). Cells were cultured for 24 h at 37 °C with 6% CO_2_ in untreated tissue culture plates before being harvested, resuspended at 5 × 10^5^/ml in viral supernatant supplemented with cytokines (see above) and incubated (3 ml/well) in Retronectin (Takara Bio Inc) coated plates (12.5 μg/ml overnight at 4 °C, unbound sites were blocked with 2% BSA for 30 min at RT) for 17 h at 37 °C with 6% CO_2_.

Cells were harvested (adherent cells were harvested using 0.25 ml of PBS-based cell dissociation buffer, Gibco, Invitrogen), pooled, washed in PBS and resuspended at 5 × 10^6^/ml in PBS with 10% FBS. The cell suspension (300 μl) was then injected into the tail vein of a Bl-6 mouse 24 h post-irradiation (2 × 500 rad (5Gy) separated over 3 h). To minimize infection mice were housed under IVC conditions and kept on 80 mg/ml neomycin for 4 weeks post irradiation. Four weeks after injection blood was collected via tail bleeds and reconstitution was assessed using FACS.

#### 3.3.4 eGFP-PH-PKB neutrophil sample preparation for electron microscopy (EM)

Cells were fixed with 8% paraformaldehyde/0.2% gluteraldehyde in 0.2 M PIPES pH 7.2, incubated and pelleted.

Neutrophils were pre-incubated with wortmannin (100 nM) or vehicle control (DMSO, 0.1%) for 5 min at 37 °C then stimulated with fMLP (10 μM). Cells were then gently vortexed and fixed with an equal volume of 8% paraformaldehyde/0.2% gluteraldehyde in 0.2 M PIPES pH 7.2. Cells were incubated at RT for 5 min, micro-centrifuged at 15,000× g for 10 min at 4 °C and then incubated at RT for 45 min, fixative was aspirated and cell pellets washed twice with PBS and stored at 4 °C.

Neutrophil samples were embedded in pig skin gelatine (PSG) by aspirating PBS from the pellets, adding 500 μl PSG, incubating for 1 h at 37 °C and then cooling on ice for 20 min, all following steps were carried out at 4 °C. Pellets were removed from the Eppendorf tube, cut into smaller pieces and transferred to an Eppendorf containing 2.1 M sucrose/PBS and incubated overnight. The following day cell blocks were prepared by cutting pellets into smaller fragments and mounting onto iron panel pins (stored in acetone). Excess sucrose was removed and pyramid shaped blocks were formed with filter paper before they were frozen and stored in N_2_ (l). Ultrathin cryosections (80 nm) were cut from frozen blocks on a Leica EM FCS ultracut UCT microtome at −110 °C using a diamond knife. Cryosections were collected from the microtome on a frozen droplet of 2.3 M sucrose/2% methylcellulose (1:1) on a wire loop and allowed to thaw before being transferred onto Pioloform (polyvinyl butyral)/carbon-coated 100 mesh hexagonal support grids. The grids were transferred to 0.1 M NH_4_Cl (in PBS) for 10 min before washing with PBS and blocking in 0.5% fish skin gelatine (FSG) in PBS for 10 min. After incubation with anti-GFP for 1 h the cryosections were washed with PBS. Bound probe was detected by incubation with 10 μl of 10 nm-particle-diameter Protein A-Gold (1:50 in FSG) for 20 min. Grids were washed in PBS and distilled H_2_O before being coated with 2% methyl cellulose/3% uranyly acetate. Grids were dried overnight and sections viewed on a JEOL 1200EX electron microscope at 80 kV.

## 4 Results

**Fig. 1:**
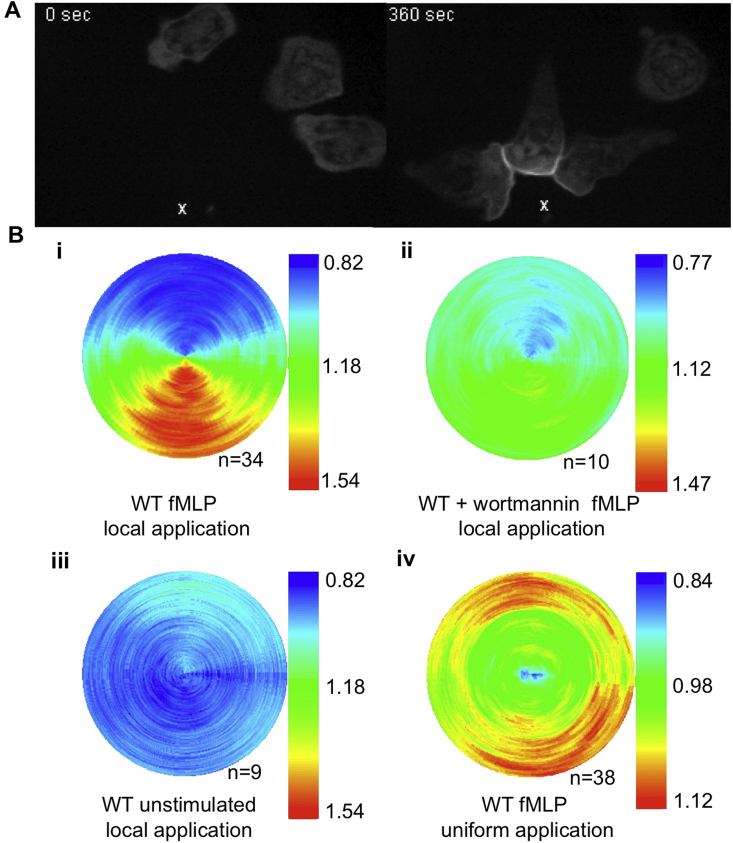
Localising Class I PI3K activity in neutrophils with a PH-domain reporter. (A) An fMLP gradient was generated with a micropipette positioned about 20 μm from eGFP-PH-PKB-expressing neutrophils (all of the figures are orientated so that this was vertically below the cell or polar plot), confocal imaging revealed, as described previously, accumulation of the reporter at the up-gradient leading edge. (B) Polar plots showed that, averaged across many movies (n = number of independent movies) and all of their component frames, the reporter accumulated at the leading, up-gradient edge when the micropipette contained fMLP (i), but not when the cells were pre-treated with wortmannin (ii, 250 nM), or the micropipette contained vehicle alone (iii). Uniform stimulation with fMLP resulted in spatially random translocation of the reporter (iv).

We, and others, have reported that a transgenically-expressed eGFP-PH-PKB reporter preferentially accumulated at the leading edge of mouse neutrophils migrating towards formylated bacterial peptides released from a micropipette (Ferguson et al., 2007, Nishio et al., 2007). We used cell-tracking and software designed to estimate the extent and vector of translocation of the reporter to the periphery, averaged for many cells, in comparison to DiIC16 membrane probes in the same cells. It was clear that the PH domain reporter, and hence PtdIns(3,4)P_2_/(3,4,5)P_3_, was concentrated in membrane at the leading edge, in a manner dependent on PI3Kγ. In Fig. 1 we demonstrate that the sustained, gradient-biased accumulation of the reporter at the leading edge is dependent on gradient-based stimulation and blocked by the PI3K-inhibitor wortmannin.

**Fig. 2:**
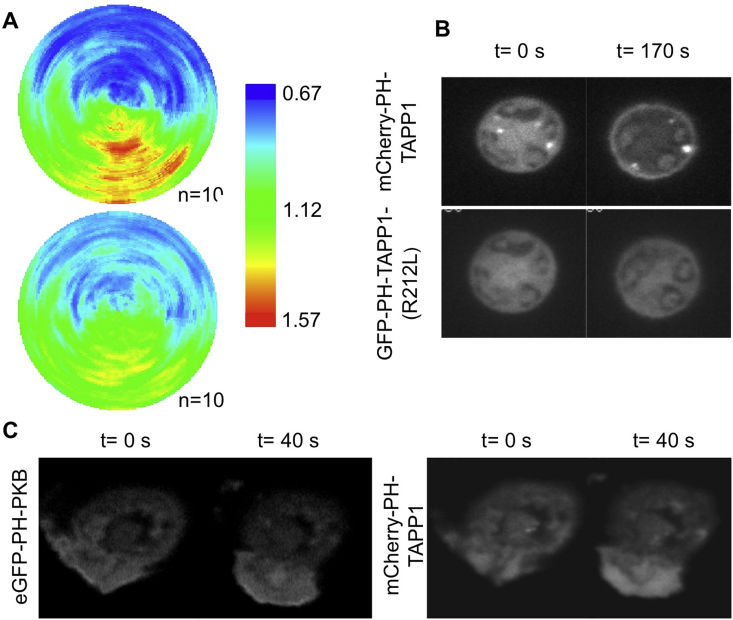
The lipid signals and morphological factors underlying polarised accumulation of PH-PKB reporters. (A) An fMLP-containing micropipette was used to stimulate neutrophils co-expressing eGFP-PH-PKB and mCherry-CAAX, polar plots were created for each reporter (eGFP-PH – upper). (B) Validation of a PtdIns(3,4)P_2_ reporter in neutrophils. Confocal images of neutrophils co-expressing mCherry-PH-TAPP-1 (upper) and eGFP-(R212L)PH-TAPP-1 (lower) stimulated with peroxy-vanadate for 0 or 170 s (representative of 4, see Fig. 3 for more frames from this movie and further controls). (C) The eGFP-PH-PKB construct reported changes in PtdIns(3,4,5)P_3_ during neutrophil chemotaxis. An fMLP-containing micropipette (position out of figure directly down) was used to stimulate neutrophils co-expressing eGFP-PH-PKB (left) and mCherry-PH-TAPP-1 (right) (representative of 3).

Expression of membrane-targeted-FP (fluorescent protein) constructs has indicated that membrane accumulation near to the leading edge can contribute to the apparent polarization of PH domain reporters (Dewitt et al., 2009, Onsum et al., 2006). We tested to what extent phenomenon impacted our observations by co-expressing of mCherry-CAAX with eGFP-PH-PKB in mouse neutrophils and then analysing their distribution during chemotaxis towards formylated peptides (Fig. 2A). Consistent with others work, the PH domain reporter was very significantly more polarized. Comparison of the distributions of the DiIC16 and mCherry-CAAX distributions indicated that the FP construct became more polarized. This could have been due to DiIC16 being excluded from, and/or mCherry-CAAX being concentrated at, the leading edge. As mCherry-CAAX is based upon a “biologically meaningful” targeting motif we assumed it represented an upper limit for potential morphological artifacts.

**Fig. 3:**
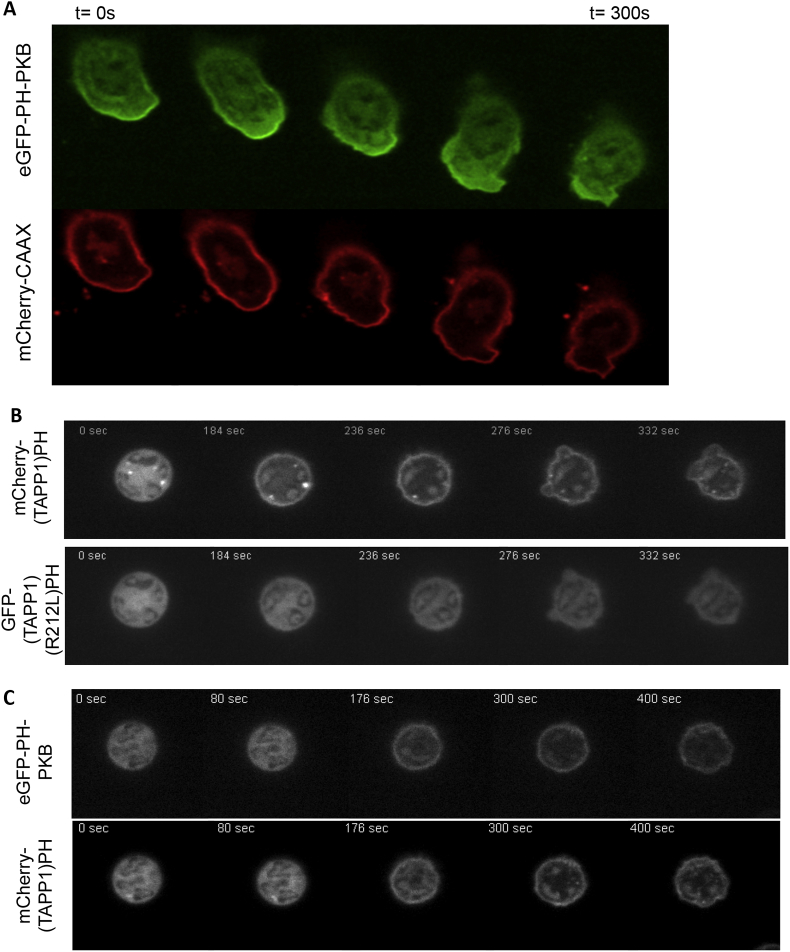
Factors underlying polarised accumulation of PH-PKB reporters. (A) An fMLP-containing micropipette was used to stimulate neutrophils co-expressing eGFP-PH-PKB (upper) and mCherry-CAAX. A representative series of frames is shown from movies analysed to produce polar plots shown in Fig. 2A and 3B. (B) Validation of a PtdIns(3,4)P_2_ reporter in neutrophils. Confocal images of neutrophils co-expressing mCherry-PH-TAPP-1 (upper panels) and eGFP-(R212L)PH-TAPP-1 (lower panels), a mutant unable to bind to PtdIns(3,4)P_2_, stimulated with peroxy-vanadate (representative of 4). (frames 1 and 2 of the series are shown in Fig. 2B) (C) Comparison of the dynamic sensitivities of the TAPP-1 and PKB-derived reporters. Neutrophils co-expressing eGFP-PH-PKB (upper) and mCherry-PH-PKB (lower) (representative of 3) were stimulated with peroxy-vanadate.

We attempted to identify the phosphoinositides that had been detected by the eGFP-PH-PKB reporter. The PH domain of PKB can bind with similar affinity to PtdIns(3,4)P_2_ and PtdIns(3,4,5)P_3_ and could have been responding to either lipid. To resolve this problem we made use of a PH-TAPP-1 construct demonstrated to be highly selective for PtdIns(3,4)P_2_ (Dowler et al., 2000, Watt et al., 2004). Co-expression of mCherry-PH-TAPP-1 with eGFP-PH-R212L-TAPP-1 (a lipid-binding mutant) in peroxy-vanadate-stimulated mouse neutrophils (this treatment drives substantial accumulation of PtdIns(3,4)P_2_ but not PtdIns(3,4,5)P_3_ (Van der Kaay et al., 1999)) showed the wild-type reporter specifically detected PtdIns(3,4)P_2_ accumulation (Fig. 2B). Co-expression of eGFP-PH-PKB and mCherry-PH-TAPP-1 in peroxy-vanadate-stimulated mouse neutrophils showed that both reporters responded similarly, both in terms of kinetics and the extent of translocation to the periphery; indicating that the dynamic sensitivity of the mCherry-PH-TAPP-1 reporter for PtdIns(3,4)P_2_ is similar to that of eGFP-PH-PKB for PtdIns(3,4,5)P_3_ (Fig. 3C). We found that fMLP-stimulation of cells co-expressing eGFP-PH-PKB and mCherry-PH-TAPP-1 repeatedly resulted in clear translocation of the former, but not the latter, reporter to the leading edge (Fig. 2C). We concluded that, despite the known role of SHIPI in mouse neutrophil chemotaxis (Nishio et al., 2007), the levels of PtdIns(3,4)P_2_ remain an order of magnitude lower than PtdIns(3,4,5)P_3_ at the leading edge and that our, and many other workers, results with a eGFP-PH-PKB reporter reflect changes in PtdIns(3,4,5)P_3_ density at the cell surface. This is consistent with measurements of these lipids by metabolic labelling with [^32^P]-Pi of fMLP-stimulated, non-adherent mouse neutrophils (Condliffe et al., 2005).

**Fig. 4:**
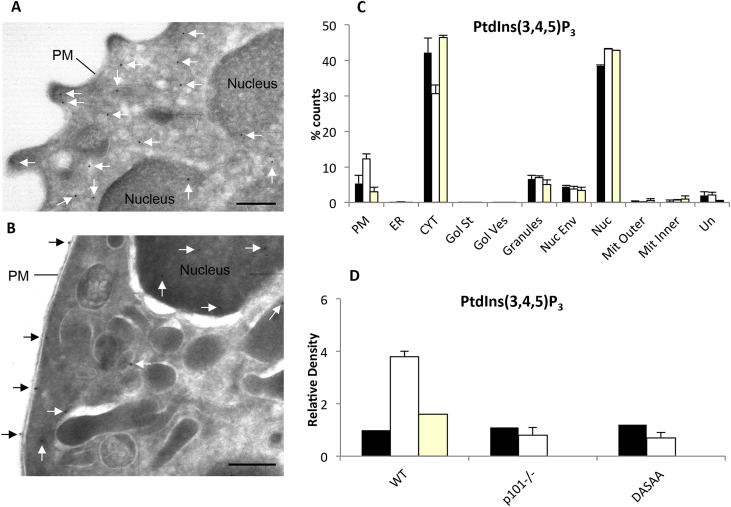
EM analysis of the localisation of eGFP-PH-PKB reporters in neutrophils. (A) Non-adherent eGFP-PH-PKB mouse neutrophils were stimulated with vehicle alone (or in panel (B) with fMLP (10 μM)) for 1 min, fixed, labelled with anti-GFP and protein-A-colloidal gold before imaging by electron microscopy. Arrows indicate the presence of gold particles and scale bars represents 200 nm (C) Non-adherent neutrophils were pretreated with wortmannin (yellow bars, 250 nM, 10 min) or vehicle (black and white bars) then stimulated with fMLP (white or yellow bars, 10 μM, 1 min) or vehicle alone (black bars), fixed, labelled with anti-GFP and protein-A-colloidal gold and visualised by EM. The number of gold particles on sampling lines were counted and binned (as % of total) based on cell morphology/organelle (PM = Plasma Membrane; ER = Endoplasmic Reticulum; CYT = Cytoplasm; Gol St = Golgi Structures; Gol Ves = Golgi Vesicles; Nuc Env = Nuclear Envelope; Nuc = Nucleus; Mit Outer = Outer Mitochondrial membrane; Mit Inner = Inner Mitochondrial membrane; Un = unassigned). Means (±range) from 2 experiments based on counting greater than 200 grids are presented. (D) Genetic loss of either Gβγ-sensitivity of PI3Kγ (p101^−/−^) or the sensitivity of p110γ to Ras (DASAA-mutant knock-in p110γ^DASAA/DASAA^) blocks the fMLP-induced rise in eGFP-PH-PKB at the plasma membrane, assay and replication as in (C). (For interpretation of the references to colour in this figure legend, the reader is referred to the web version of this article.)

The precision with which we could locate the eGFP-PH-PKB reporter, and hence the endogenous PtdIns(3,4,5)P_3_, was limited by the resolving power of light microscopy and could only be stated to be peripheral. To advance this problem we used EM techniques. To achieve this we rapidly fixed, froze and cryo-sectioned eGFP-probe-expressing neutrophils before applying an anti-GFP antibody and gold-labelled secondary antibodies. The approach avoided the challenges associated with detection of endogenous phosphoinositides by application of lipid-binding probes after fixation (Lindsay et al., 2006, Watt et al., 2004, Watt et al., 2002). We found that upon fMLP-stimulation there was a reduction in the intensity of staining of the cytoplasmic compartment (Fig. 4A and C) and a large increase in the density of decoration of the plasma membrane (Fig. 4B and C), but not other membrane fractions (Fig. 4C), that was inhibited by wortmannin, genetic loss of p101, the major Gβγ-sensitive p110γ adaptor in neutrophils, or knock-in of a Ras-insensitive version of p110γ, p110γ^*DASAA*^ (by creating the lines eGFP-PH-PKB x p101^−/−^ and eGFP-PH-PKB x p110γ^*DASAA/DASAA*^; Fig. 4C and D). This is entirely consistent with the wortmannin-sensitivity of PI3Kγ and known roles of Gβγs and Ras in its regulation and further validates the veracity of this assay.

**Fig. 5:**
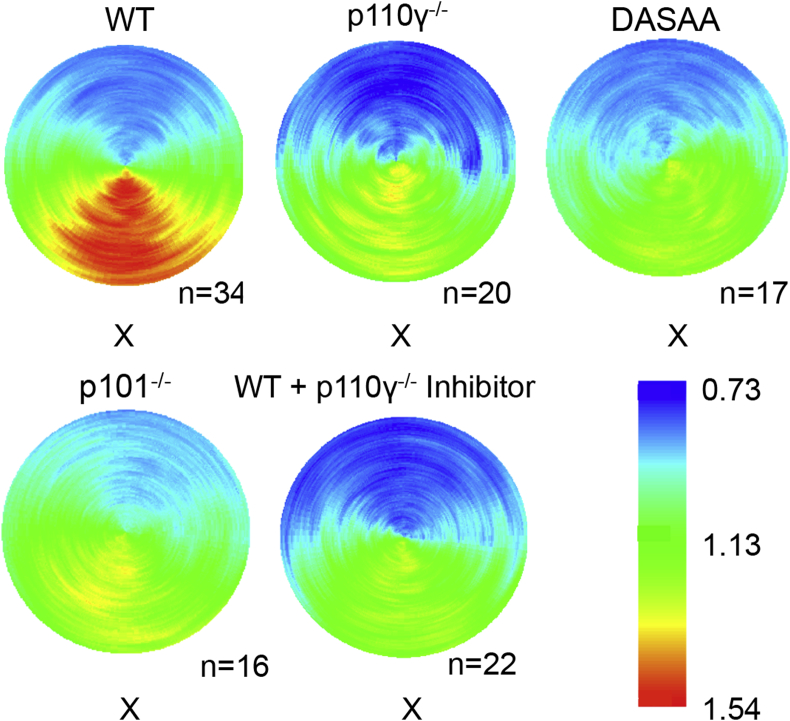
Both Gβγ and Ras regulation of PI3Kγ are crucial for accumulation of PtdIns(3,4,5)P_3_ at the leading, up-gradient edge. Polar plots of neutrophils, responding to an fMLP-containing micropipette, which expressed eGFP-PH-PKB in different genetic backgrounds (x wild type (WT); x p110γ^−/−^; x p110γ^DASAA/DASAA^; x p101^−/−^) or pre-treated with a p110γ inhibitor (AS252424; 10 μM, using x WT cells).

PI3Kγ can be regulated by both Gβγs and GTP-Ras in mouse neutrophils (Suire et al., 2006). We sought to test the idea that these inputs might contribute differentially to driving PtdIns(3,4,5)P_3_ accumulation at the leading edge using the mouse strains described above; eGFP-PH-PKB x p101^−/−^ and eGFP-PH-PKB x p110γ^*DASAA/DASAA*^. The distribution eGFP reporter was analysed in live cells chemotaxing towards fMLP using a spinning disc confocal microscope. We found that loss of p101, genetic blockade of Ras-regulation of PI3Kγ and transient chemical inhibition of PI3Kγ similarly and substantially inhibited accumulation of PtdIns(3,4,5)P_3_ at the leading edge (Fig. 5). This suggests both Gβγs and GTP-Ras are driving PI3Kγ in its roles in the leading edge of migrating neutrophils.

## 5 Discussion

Our results are broadly in keeping with the literature. We have used EM techniques to localize an endogenously-expressed eGFP-PH-PKB reporter. Our data indicated that in controls cells the reporter was in the cytoplasmic compartment. Following stimulation with fMLP the reporter localized to the plasma membrane, and not other membranes. We found no evidence for increased accumulation of the reporter in the nucleus following fMLP-stimulation of mouse neutrophils. Our results with reporters capable of sensing PtdIns(3,4)P_2_ and previous work measuring PtdIns(3,4)P_2_ accumulation both suggest that the eGFP-PH-PKB construct is being localized by interactions with PtdIns(3,4,5)P_3_. These data indicate PtdIns(3,4,5)P_3_ accumulates in the plasma membrane; they do not, however, demonstrate that PtdIns(3,4,5)P_3_ only rises in the plasma membrane. There is evidence that PH domains contain motifs that bind to specific types of cell membrane and that these interactions, in addition to interactions between phosphoinositides and the PH domain, are required to allow membrane recruitment (Hammond and Balla, 2015). As a result PtdIns(3,4,5)P_3_ could accumulate in membranes other than the plasma membrane but would not be sensed by PtdIns(3,4,5)P_3_-binding PH domains. It is unclear if domains that bind PtdIns(3,4,5)P_3_ that are not PH domains have the same properties.

The TAPP1 construct we used to sense PtdIns(3,4)P_2_ was capable of reporting an increase in PtdIns(3,4)P_2_ in peroxy-vanadate-stimulated neutrophils. We could not find any evidence of fMLP-induced localization of the reporter to the leading edge. These results do not allow us to conclude there is no PtdIns(3,4)P_2_ accumulation at the periphery of fMLP-stimulated neutrophils, indeed much evidence shows PtdIns(3,4)P_2_ does accumulate in stimulated neutrophils (Stephens et al., 1991, Traynor-Kaplan et al., 1989), but rather the concentrations achieved are insufficient to relocate a significant proportion of the reporter. As a result it is unclear if the distribution of that PtdIns(3,4)P_2_ had merely followed PtdIns(3,4,5)P_3_ or not.

There is evidence that p84-PI3Kγ drives accumulation of PtdIns(3,4,5)P_3_ and/or PtdIns(3,4)P_2_ in different near-plasma membrane locations to p101-PI3Kγ in mast cells (Bohnacker et al., 2009). We attempted to address this question in neutrophils. Neutrophils contain significant amounts of both p84 and p101-PI3Kγ complexes. In the experiments reported here we could find no evidence of PtdIns(3,4,5)P_3_ accumulation in the plasma membrane as detected by EM-analysis when neutrophils only contain p84-PI3Kγ (ie in p101^−/−^ neutrophils) or in cells in which PI3Kγ could not be activated by Ras but could be activated via p101 Gβγs (p110γ^DASAA/DASAA^). These results precluded an analysis of the above problem and are surprising as experiments measuring the amount of PtdIns(3,4,5)P_3_ in neutrophils of these genetic backgrounds indicated the responses to fMLP were only partially reduced and not abolished. Perhaps the EM-based methodology leads to a highly non-linear relationship between staining and PtdIns(3,4,5)P_3_ content or, alternatively, that a significant amount of the PtdIns(3,4,5)P_3_ we have measured is localized in a non-plasma membrane location that we cannot observe/recover. We also compared the localization of an eGFP-PH-PKB reporter in living p101^−/−^ and p110γ^DASAA/DASAA^-expressing neutrophils. We found no evidence that the reporter localized differently in these different genetic backgrounds although our result suggested both had reduced accumulation of PtdIns(3,4,5)P_3_ at the leading edge compared to wild-type neutrophils. These experiments, however, relied upon either light microscopy and hence had limited spatial resolution. Hence it remains possible that there are spatiotemporal differences in the PtdIns(3,4,5)P_3_ accumulated following Gβγ v Ras-activation of PI3Kγ but that we failed to detect them through limitations in our methodologies.

## Conflicts of interest

The authors declare no conflicts of interest.
